# The effectiveness of behavioural interventions in the primary prevention of Hepatitis C amongst injecting drug users: a randomised controlled trial and lessons learned

**DOI:** 10.1186/1477-7517-5-25

**Published:** 2008-07-31

**Authors:** Mohammed Abou-Saleh, Paul Davis, Philip Rice, Ken Checinski, Colin Drummond, Douglas Maxwell, Christine Godfrey, Christopher John, Betsy Corrin, Christopher Tibbs, Adenekan Oyefeso, Marian de Ruiter, Hamid Ghodse

**Affiliations:** 1 Division of Mental Health, St. George's, University of London, Cranmer Terrace, Tooting, London, SW17 0RE, UK; 2 Substance Misuse Services, Camden and Islington NHS Foundation Trust, London, UK; 3 Department of Health Sciences, The University of York, York, UK; 4 Addictions, Surrey and Borders Partnership NHS Foundation Trust, Chertsey, Surrey, UK; 5 Department of Gastroenterology, Royal Surrey County Hospital, Guildford, UK

## Abstract

**Aim:**

To develop and evaluate the comparative effectiveness of behavioural interventions of enhanced prevention counselling (EPC) and simple educational counselling (SEC) in reducing hepatitis C viral (HCV) infection in sero-negative injecting drug users (IDU).

**Design:**

Randomised controlled trial (RCT) of EPC intervention in comparison with simple educational counselling (SEC).

**Setting Specialised:**

Drug services in London and Surrey, United Kingdom.

**Participants and Measurements:**

Ninety five IDUs were recruited and randomised to receive EPC (n = 43) or SEC (n = 52). Subjects were assessed at baseline using the Addiction Severity Index (ASI), the Injecting Risk Questionnaire (IRQ), and Drug Injecting Confidence Questionnaire (DICQ). The primary outcome was measured by the rate of sero-conversion at 6 months and 12 months from baseline and by the ASI, IRQ and DICQ at 6 months from baseline. Hepatitis C testing was undertaken by the innovative test of the dried blood spot (DBS) test which increased the rate of testing by 4 fold compared to routine blood testing.

**Findings Seventy:**

Eighty two subjects (82%) out of the 95 recruited were followed up at 6 months and 62 (65%) were followed up at 12 months. On the primary outcome measure of the rate of seroconversion, 8 out of 62 patients followed-up at twelve months seroconverted, three in the EPC group and five in the SEC group, indicating incidence rates of 9.1 per 100 person years for the EPC group, 17.2 per 100 person years for the SEC group, and 12.9 per 100 person years for the cohort as a whole. Analysis of the secondary outcome measures on alcohol use, risk behaviour, psychological measures, quality of life, showed no significant differences between the EPC and the SEC groups. However, there were significant changes on a number of measures from baseline values indicating positive change for both groups.

**Conclusion:**

We were not able to prove the efficacy of EPC in comparison with SEC in the prevention of hepatitis C in IDUs. This was related to low recruitment and retention rates of the participants. Moreover there was a low adherence rate to EPC. The study provided the benefits of developing and introducing behavioural interventions of the EPC and SEC and the DBS screening for Hepatitis C. Moreover the main lessons learnt were that piloting of a new intervention is a crucial first step before conducting pragmatic RCTs of psychological interventions in the field of addiction; that an infrastructure and culture for psychosocial interventions is needed to enable applied research in the service environment, and research funding is needed for enabling the recruitment of dedicated trained therapists for the delivery of these interventions.

## Background

Viral hepatitis C is a global public health problem, and has been considered one of the major challenges in the third millennium [1]. Injecting drug use is the main route of transmission, mediated by the sharing of injection equipment, especially needles and syringes but also spoons, cotton filters and other paraphernalia [2]. In the UK, studies of prevalence rates of anti-HCV amongst Injecting Drug Users (IDUs) reported rates of 80%–86% in England (5 studies) and 37%–90% in Scotland (3 studies) [3]. The association between injecting drug use and infection with HCV is mediated by sharing needles and syringes or other injecting paraphernalia. European studies showed rates of sharing needles and syringes and other injecting paraphernalia between 70% and 94% [4].

The recently introduced Hepatitis C Strategy for England [5] laid strong emphasis on preventing new cases of hepatitis C infection in IDUs by health promotion activities and the provision of needle exchange schemes. This is best achieved in the context of treatment for drug dependence complemented with information about hepatitis C and harm minimisation messages. However, this new policy falls short of recommending specific preventive interventions which are evidence based; hence the importance of this project which aims to evaluate a new preventive intervention for hepatitis C in IDU's.

The aim of the present study was to evaluate the effectiveness of enhanced prevention counselling (EPC) in reducing HCV infection in HCV sero-negative patients. Our primary hypothesis was that EPC is more effective and cost-effective than simple educational counselling (SEC) in reducing the rate of HCV sero-conversion and its risk behaviour. Whilst we have also evaluated sexual risk behaviour in relation to the occurrence of HCV, we have not studied the prevalence and seroconversion rates of hepatitis B, HIV and other sexually transmitted diseases.

However we were not able to prove the efficacy of EPC in comparison with SEC in the prevention of hepatitis C in IDUs. This was related to low recruitment and retention rates of the participants. Moreover there was a low adherence rate to EPC.

In view of these reported negative findings, we have also provided an overview of the main problems that we faced and our attempts to overcome them, in the hope that it will guide future researchers in the field of prevention interventions in addiction [6].

## Methods

The study was conducted in 2 phases, a screening phase and an intervention phase.

### Screening phase

Injecting drug users presenting to collaborating drug treatment services in South West London, North London and in Surrey were screened for eligibility in four steps: (1) the identification of IDUs, (2) distinguishing between those IDUs that have injected at least once in the last six months and those that have not, (3) assessment of IDUs for inclusion and exclusion criteria and (4) eligibility by testing for current HCV sero-positivity using a standard ELISA HCV antibody test [7]. All IDUs who were confirmed to be HCV sero-negative by HCV antibody test were invited to attend a research interview conducted by the research workers. This occurred in the context of their ongoing care. Successive IDUs referred to community drug services were recruited to the trial as well those who were in treatment. The overall retention in treatment rate during the trial was 60% with no difference in retention rates between those receiving EPC and SEC.

#### Inclusion Criteria were

(1) male and female IDUs (2) age 18–70 years (3) ICD-10 diagnosis of mental and behavioural disorder due to the use of drugs (F11-F19) established by baseline research interview (4) willingness to nominate an independent informant to provide collateral information, and nominate a locator who can assist in tracing the subject at follow-up (5) stable place of residence (defined as having a domicile for which there is no imminent danger of eviction) and (6) living close enough to commute to the clinic.

#### Exclusion Criteria were

(1) current severe mental illness (e.g. bipolar effective disorder or schizophrenia), (2) severe physical illness that would preclude participation, (3) serious legal problems, including impending imprisonment, likely to interfere with treatment participation and/or follow-up and (4) severe brain damage or mental impairment. The inclusion and exclusion criteria were established by a combination of clinical assessment by service staff, and baseline research interview conducted by the research workers.

Ethical approval for the research was sought and obtained from both the Multi-Centre Research Ethics Committee (MREC) and relevant Local Research Ethics Committees (LREC) where recruitment took place. The recruitment process, issues of information provision, consent, confidentiality, data protection, management of the research, and all other aspects of the Trial were modelled on the recommendations for good research and clinical practice provided by the MREC and LREC guidelines.

#### Baseline assessment

All drug users were assessed using the European Addiction Severity Index [8], Injecting Risk Questionnaire (IRQ) [9], the HIV Risk Taking Behaviour Scale [10] and Alcohol Use Disorders Identification Test (AUDIT) Questionnaire [11]. Self-efficacy, outcome expectancies (situational confidence) were measured using an adapted version of the Situational Confidence Questionnaire [12]. Finally, stages of change in the "readiness to change" model [13] were assessed using the Readiness to Change questionnaire, and general knowledge on hepatitis C measured using a custom-designed questionnaire.

### Intervention phase

After the completion of a baseline assessment, all clients were randomised to receive either the Enhanced Prevention Counselling (EPC) or Simple Educational Counselling (SEC) intervention. The therapists who administered the interventions were regular staff of community drug services that took part in the trial. All therapists received an intensive training programme in the administration of manualized EPC and SEC from PD who is an accredited clinical psychologist with national expertise in training clinicians in psychological interventions. The therapists received regular supervision from PD and other trained supervisors who completed the intensive training in EPC intervention and the techniques of supervision from PD. For quality control, all sessions were audio-taped to measure the fidelity of the EPC and SEC interventions.

#### Enhanced prevention counselling

This comprised four sessions of manual-guided intervention. The manual was based on a number of other treatment intervention manuals, and particularly on the Brief Intervention (Motivational Enhancement Therapy) used in Project Match (Project MATCH Research Group, 1998), and the manual established and evaluated for project RESPECT which was concerned with the reduction of high-risk sexual behaviour and the introduction of safer sex [14]. In addition, exercises and elements were taken from substance misuse cognitive behavioural treatments [15], and elaborated in therapy manuals [16,17].

The four sessions were intended to be completed within eight weeks of entry into the programme and were carried out by a drug clinic worker who was trained in delivering the intervention but who was not involved in collecting outcome data from participants. The aim of the intervention was to reduce risk behaviours associated with the acquisition of HCV infection in injecting drug users. HCV transmission risk behaviours include injecting drugs, the sharing of injecting equipment, not cleaning and reusing drug paraphernalia, alcohol misuse, cocaine use, unprotected sexual activity, multiple sexual partners, and non-compliance with methadone treatment.

EPC as applied in this project utilises principles of motivational psychology, theories of behaviour change (particularly social cognitive learning theory), and health belief models, including the theory of reasoned action [18,19]. Changes in risk behaviour are hypothesised to take place through changes in outcome expectancies (expected consequences of a course of action, e.g. sharing injecting equipment) and self efficacy (confidence in one's ability to achieve a particular goal, e.g. avoidance of sharing injecting equipment). Motivational interventions have been applied to a variety of health behaviours in addition to addictive behaviour (reviewed in Miller and Rollnick [20]; [21], and can be readily applied to health promotion in drug misusers. The aim of the intervention is to enhance the subject's self-perception of risk and facilitate the development of individual strategies to avoid engaging in HCV risk activities. The measurement of self-efficacy will allow assessment of the process of the intervention.

All sessions last between 40–60 minutes and follow the format of the brief interventions described above. Session one has the aim of establishing rapport and a counselling relationship consistent with the principles of Motivational Interviewing, increasing knowledge and awareness of HCV risk behaviours and the consequences of HCV infection, introducing the rationale and structure of the intervention to the subject, and assessing the individual's risk behaviours, self efficacy and outcome expectancies. The remaining three sessions begin with a review of progress on targets set at the previous session, assessment of any difficulties in applying coping strategies, and assessment of the subject's motivational state. In the second session, if appropriate, targets for intervention are planned (e.g. unprotected sexual activity, injecting equipment sharing). Each session ends with a behavioural goal-setting exercise in which the participant arrives at a small behavioural risk-reduction step that could be achieved by the next session. At the end of the final session, a longer-term, individualised risk reduction plan is agreed upon. Various strategies are employed in EPC to foster compliance with the intervention, including the development of a therapeutic alliance, individual risk reduction plans to take home as a reminder of behavioural goals, appointment cards, combining sessions with regular clinic visits (e.g. for methadone prescriptions), and phoning the subject on the day of visits as a reminder.

#### Simple Educational Counselling (SEC)

This consisted of a ten-minute session of information-giving intervention about the nature and the risk factors of HCV, with advice on prevention including the need to reduce sharing of injecting equipment and safer injecting practices. It was intended to be a non-interactive intervention in order to contrast with the EPC, and clients were asked to direct any questions they might have to their key worker rather than the counsellor.

### Outcome Measurement

Research follow up interviews were conducted at six months post randomisation, and blood tests for hepatitis C at both six months and twelve months. The primary outcome measure was the number of new cases of HCV infection by sero-conversion detected by HCV positive antibody at 6 and 12 months from recruitment. Secondary outcome measures were those administered at baseline.

### Sample size

Power calculations were based upon rates of sero-conversion of 6% per hundred person years found in a research study that applied intensive preventative counselling to IDUs [22]. This was compared with the rate of sero-conversion obtained from research into the IDU population of 20% per hundred person years [23]. Based on these figures, the difference was estimated to be around 14%, and so with a power of 0.8 and a difference proven at the 5% level using a two-tailed test, a followed-up sample of 180 IDUs was required.

### Randomisation

Randomisation was stratified by two variables in order to provide a control for what were perceived to be potentially important influences. The stratification variables were the "Treatment Centre" that the client was recruited from, to control for differences in standard service at each of the recruitment sites, and "Injecting Equipment Sharing Behaviour", to control for a very important risk-factor predictor for contracting hepatitis C. Stratification was achieved in blocks of six within each variable, such that in each block of six half of the clients would receive SEC and half of the clients EPC. This method of stratified randomisation was chosen over true randomisation in order to ensure that allocation to either group was fairly constant throughout the life of the trial, helping to maximise therapist time by spreading the workload more evenly. It was also intended to act as a safeguard against bias to one intervention or the other if lower than expected levels of recruitment were achieved.

The physical implementation of the randomisation scheme was accomplished by a custom-designed statistical computer program which generated stratified random integers between 1 and 2 in blocks of six; these were then transcribed onto cards and sealed in envelopes by a person unconnected with the Research Team, so that they never had any access to the randomisation scheme used. Envelopes were marked sequentially on the outside, and were opened by the Research Team upon completion of a baseline interview. Other features which contributed to the protection of the validity of the Trial and maximising the quality of the data included the written protocol that was followed, the manual-guided treatment interventions, and quality control of the interventions through the rating and assessing of a random sample of tapes from the sessions.

### Blinding

Although it would have been preferable for the purposes of completely eliminating the potential for bias to make the Research Team blind to the therapy intervention allocated, due to the Research Team's need to co-ordinate and help facilitate the implementation of the intervention this was not possible. Once the therapy allocation was made, the Research Team had to locate a suitable therapist to conduct the intervention and liaise with them over the progress that they were making with the clients allocated to them, and this applied to both EPC and SEC therapists. Arrangements for the payment of travel expenses for clients attending the EPC sessions also had to be made (sometimes for both client and therapist), and the researchers also played a large role in chasing up clients who did not attend their sessions. Thus, although it may have been possible to implement a blinding procedure, it was felt that the benefits of doing so were outweighed by the increases in efficiency gained otherwise. It was also felt that not blinding would have minimal impact on the research outcomes as the primary outcome measure was rate of sero-conversion, which is not open to bias, and in addition majority of the interview measures were direct and quantitative rather than subjective and qualitative.

### Statistical Analyses

Data analysis was conducted on intention to treat basis. The primary hypothesis was tested using chi-square for comparison of rates of sero-conversion and using analysis of covariance with risk-behaviour composite scores as dependent variables, controlling for baseline values on these measures, and treatment condition as the independent variable. Similar analysis was carried out on secondary outcome measures with calculations of differences between intervention groups and differences between the group who were and the group who were not followed up. Incidence of HCV was calculated using the person years method [24] among IDUs who were sero-negative for HCV antibody and who had repeated testing after 6 and 12 months from baseline testing. Analyses of covariance were performed for all relevant secondary outcome measures, using baseline scores as the first covariate to control for initial individual differences, and baseline score on the injecting subscale of the HIV Risk-Taking Behaviour Scale as the second covariate where appropriate, as this subscale was identified as being significantly different between the two interventions. Chi-square analyses were performed for all categorical data and Mann-Whitney U-Tests for ordinal data that were not normally distributed to apply parametric tests.

#### Dried Blood Spot (DBS) testing for hepatitis C

When recruitment began, it was found that far less testing of hepatitis C was happening at community drug teams than was reported, and this proved to be a major obstacle for the trial. It was suggested this could be overcome with the implementation of the Dried Blood Spot (DBS) test [25] for hepatitis C, and after seeking approval from the Department of Health and the Trial Steering Committee the procedure was adopted at all recruitment centres. A study into the validity of the DBS test revealed it to have 100% sensitivity and 100% specificity [26], and our own piloting work confirmed this. The introduction of the DBS test increased testing more than fourfold, greatly assisting the recruitment process.

## Results

### Baseline analysis

#### Participants

**Figure 1:**
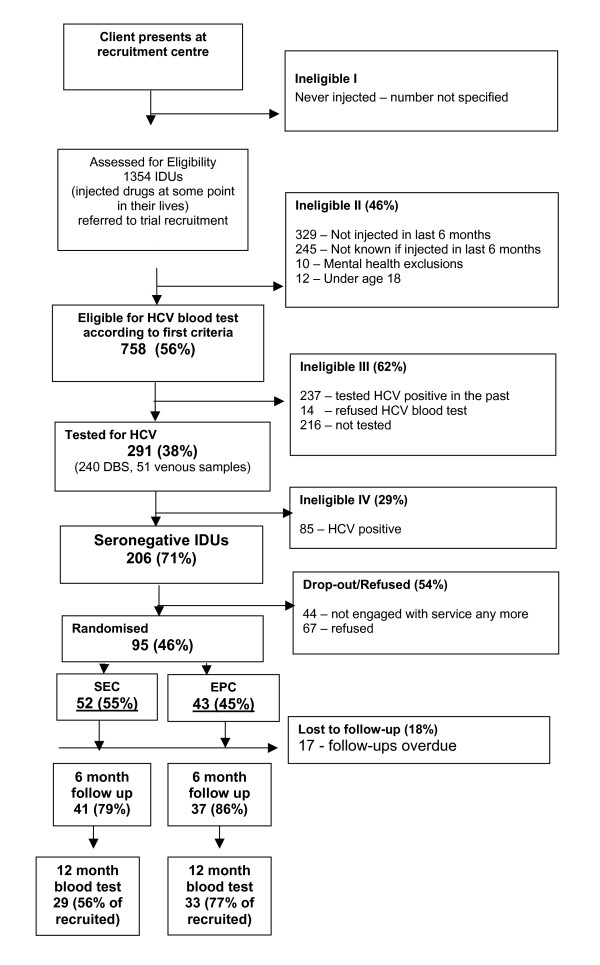
Number of participants at each stage of the recruitment process.

A flow diagram detailing the number of IDUs at each stage of the recruitment process is presented in figure 1. As shown 95 subjects were recruited and 78 were followed up at 6 months and 62 were followed up at 12 months.

#### Demographic characteristics

The mean age of all those recruited was 32 years (SD 6.7). There were 70 males, 25 females, 10% were married, 42% had at least one child, 43% were unemployed and 48% had educational qualifications. There were no significant differences on these basic demographic characteristics between both those followed-up and those not followed-up and between those allocated to EPC and those allocated to SEC.

#### Drug use and other characteristics

Participants showed the following drug use characteristics (means and SDs): duration of drug use (11.4, 7.6 years), age of first drug injection (24.5, 6.3 years), duration of injecting drug use (5.9, 4.8 years), previous episodes of treatment (3.1, 2.9) and inpatient treatment episodes (1.8, 3.1). Every recruited client was currently receiving a prescription for a substitute drug with methadone being the most commonly reported drug at 85%, with the remainder prescribed buprenorphine. One of the main criteria for recruitment was having injected at least once in the past six months, but 49% of respondents reported having injected in the last thirty days. The vast majority were regular smokers of cigarettes (89%), and 70% drank alcohol at least once per week, 35% reported that a member of their immediate family had a history of alcohol problems, 31% reported a family history of drug problems, and 28% reported a family history of psychiatric problems, 41% reported a history of emotional abuse by significant others, 23% reported a history of physical abuse, and 11% reported incidences of sexual abuse.

There were no significant differences between those followed-up and those not followed-up, or between those allocated to either intervention, on any of these measures.

#### Standardised measures

On the ASI just under 56% of clients had shared an item of injecting equipment in the last six months, with the mean number of people that they had shared with being 1.84 for the subgroup of those who admitted to any sharing at all, or 1.08 overall. Scores on the HIV Risk-Taking Behaviour Scale were expectedly high, particularly for injecting risk (mean 8.8, SD 5.7) as opposed to sexual risk (mean 4, SD 4.1), 37% were identified as having a probable drink problem by the AUDIT and 54% had used a needle exchange at least once in the last six months.

The only significant differences on all measures between those followed-up and those not followed-up were on the legal and economic subscales of the ASI, with those not followed up exhibiting relatively higher degrees of problem on both measures than those followed-up. There was one significant difference between the intervention groups, with those allocated to EPC scoring more highly on the injecting subscale of the HIV Risk-Taking Behaviour Scale, although not scoring significantly differently overall. As this subscale is quite important, indicating a higher level of sharing behaviour that could have an impact on the outcome variables and the trial intervention, this measure was used as a covariate where appropriate in the outcome analysis.

#### Psychological Measures

Confidence at resisting the urge to inject, measured by the Drug Injecting Confidence Questionnaire, was on average 59%, but large variations were noted across subjects. Unpleasant emotions, urges and temptations, and social pressure to use were areas where respondents were least likely to resist the urge to inject, whilst circumstances of pleasant emotions was the area where patients were most confident that they would not inject. Knowledge of hepatitis C, as measured by our item true-or-false questionnaire was better than expected, with average scores of over 16 out of twenty. "Stage of change", measured by the Readiness to Change Questionnaire, revealed that the majority of subjects were at the "Action" stage of change, probably reflecting the locations from which they were recruited. There were no significant differences on these measures between those followed-up and those not followed-up, or between those allocated to either of the trial interventions.

### Outcome analysis

As shown in Figure 1, out of 95 participants recruited, 78 (82%) were followed up at 6 months and 62 (65%) were followed up at 12 months. These follow up rates do not correspond to retention rates in treatment as some of the participants had dropped out from treatment but agreed to attend for the follow-up research interview and HCV testing. Moreover for SEC, 41 (79%) attended at 6 months and 29 (56%) at 12 months whilst for EPC, 37 (86%) attended at 6 months and 33 (77%) at 12 months.

Table 1 illustrates the number of participants who engaged for their allocated intervention, defined as either completing the SEC intervention or attending for at least one session of EPC of those followed-up. Table 2 illustrates the number of completed sessions of EPC.

**Table 1:** Participants engaged, not engaged, sero converted and incidence of HCV in EPC and SEC groups

	**Number** ** Engaged^a^**	**Number** ** Not engaged**	**Number** **Sero** ** converted**	**Total**	**Incidence of HCV ** ** (per 100 person years)**
**EPC**	17* (45.9%)	20 (54.1%)	3 ^b^	37	9.1
**SEC**	38 (92.7%)	3 (7.3%)	5 ^c^	41	17.2
**Total**	55 (70.5%)	23 (29.5%)	8	78	12.9

* Chi-squared test χ^2 ^= 20.43, p < 0.000

(EPC) Enhanced Prevention Counselling

(SEC)Simple Education Counselling

a Number of participants completing EPC sessions: none 20; one session 6; two sessions 4; three sessions none and four sessions 7.

b Three participants seroconverted at 12 months: none engaged with EPC: HCV incidence 9.1 per100 person years

c Five particpants seroconverted at 12 months: 4 engaged with SEC: HCV incidence 17.2 per 100 person years

**Table 2:** Number of participants who completed or have not completed EPS and the number of sessions completed

**Number of** ** EPC Sessions**	**None**	**One**	**Two**	**Three**	**Four** **(course** ** completed)**
Number of participants	20	6	4	0	7

There was a significant difference between the two groups in terms of their engagement with the therapy intervention (p < 0.000). 78 participants were followed-up at six months (82.1%), and 62 at 12 months (65.3%). Overall, six-month follow-up data indicated a seroconversion rate of 9.0% in six months, or 18.0% per year. Twelve-month follow-up data indicated a seroconversion rate of 12.9% per year.

The difference in seroconversion was not significant between the two interventions at either six months or twelve months, but it was however in the anticipated direction, with fewer of those allocated to EPC seroconverting compared to those that received SEC. The difference was even more pronounced (but still not significant) when only those who received at least one session of the intervention were included as no patients who received at least one session of EPC seroconverted at either six months or twelve months.

There were no significant differences between the EPC and SEC groups on any of the secondary outcome measures (effect of treatment). However there were significant changes in a number of measures for both groups at 6 months follow-up (effects of time). Table 3 shows significant changes for ASI alcohol use, medical subscale, economic subscale, satisfaction subscale and HIV-RTBS injecting risk, sexual risk behaviour and overall scores.

**Table 3:** Changes in addiction severity and risk behaviour

**Drug and Alcohol**	**Baseline**				**Six-Month follow-up**							
	**EPC n = 37**		**SEC n = 41**		**EPC n = 36**		**SEC n = 41**		**Effect of ** **Treatment**		**Effect of** ** Time**	

**ANCOVA**	**Mean**	**sd**	**Mean**	**sd**	**Mean**	**sd**	**Mean**	**sd**	**F**	**p**	**F**	**p**

ASI – drug use	0.286	0.12	0.29	0.096	0.22	0.152	0.249	0.133	0.90	0.35	0.54	0.47
ASI – alcohol use	0.084	0.17	0.115	0.176	0.113	0.176	0.082	0.143	2.26	0.14	41.08	0.00
ASI – medical subscale	0.138	0.26	0.134	0.278	0.092	0.207	0.156	0.294	0.95	0.33	10.97	0.00
ASI – psychiatric subscale	0.208	0.26	0.174	0.204	0.204	0.241	0.159	0.207	0.34	0.56	3.74	0.06
ASI – Legal subscale	0.094	0.16	0.119	0.182	0.093	0.151	0.09	0.177	0.01	0.92	0.80	0.38
ASI – economic subscale	0.877	0.32	0.652	0.432	0.765	0.357	0.766	0.381	0.83	0.67	8.56	0.01
ASI – satisfaction subscale	0.289	0.31	0.235	0.27	0.222	0.334	0.154	0.266	0.65	0.42	5.72	0.02
ASI – family relationships	0.101	0.16	0.13	0.209	0.075	0.133	0.077	0.151	0.02	0.89	2.31	0.13
ASI – social relationships	0.104	0.19	0.085	0.133	0.03	0.102	0.039	0.084	0.13	0.72	2.07	0.16
IRQ – No. people shared IV equipment with in last 6 months	0.81	0.88	1.32	1.4	0.22	0.64	0.37	0.97	1.1	0.31	0.00	0.98
HIV RTBS – Drug score	9.95	5.74	8.02	5.8	3.31	5.3	3.4	5.6	0.4	0.55	10.0	0.00
HIV RTBS – Sex score	4.5	4.2	3.1	3.4	4.7	4.2	3.9	4.14	0.1	0.78	8.2	0.01
HIV RTBS – Overall	13.8	7.45	11.2	6.98	8.0	8.12	7.3	6.86	0.0	0.84	11.4	0.00

Table 4 shows non significant reduction in injecting behaviour, sharing, use of needle exchange and AUDIT in the last 6 months.

**Table 4:** Changes in risk behaviour and alcohol misuse

**Drug and Alcohol**	**Baseline**				**Six-Month follow-up**					
	**EPC n = 37**		**SEC n = 41**		**EPC n = 36**		**SEC n = 41**		**Effect of** ** Treatment**	

χ^2 ^**analysis**	**N**	**%**	**N**	**%**	**N**	**%**	**N**	**%**		**p**

IRQ – injected at all in last six months	37.0	100.0	41.0	100.0	20	55.6	24	58.5	0.07	0.79
IRQ – Shared any IV equipment at all in last 6 months	21.0	56.8	27.0	65.9	6	16.6	8	19.5	0.10	0.75
Used needle exchange in the last six months	21.0	56.8	23.0	56.1	16	44.4	17	41.5	0.07	0.79
AUDIT (score of 8 or more)	11.0	29.7	16.0	39.0	8	22.2	7	17.1	0.32	0.57

Table 5 shows significant changes in all DICQ scales indicating moderate increases in situational confidence in the ability to resist the urge to inject and increases in Hepatitis C knowledge questionnaire.

**Table 5:** Changes in situational confidence, hepatitis C knowledge and readiness to change measures

**Measure**	**Baseline**				**Six-month Follow-up**							
	**EPC n = 37**		**SEC n = 41**		**EPC n = 36**		**SEC n = 41**		**Effect of ** **Treatment**		**Effect of ** **Time**	

**ANCOVA**	**Mean**	**sd**	**Mean**	**sd**	**Mean**	**sd**	**Mean**	**sd**	**F**	**p**	**F**	**p**

DICQ – Unpleasant emotions	49.27	30.1	54.51	30.06	56.86	28.8	62.85	31.56	0.38	0.54	14.44	0.00
DICQ – Physical discomfort score	56.76	27.2	61.9	28.26	64.11	24.09	66.27	30.85	0.01	0.92	10.07	0.00
DICQ – Pleasant emotions	74.16	25.3	79.17	21.67	77.44	24.62	78.51	25.73	0.03	0.87	15.38	0.00
DICQ – Testing personal control	57.76	30.7	60.44	31.51	64.53	28.76	63.1	32.42	0.05	0.82	11.54	0.00
DICQ – Urges and temptations	49.27	30.2	56.98	29.4	58.22	28.41	59.61	31.64	0.06	0.81	15.08	0.00
DICQ – Conflict with others	59.27	30.3	65.24	28.36	67.61	26.35	68.59	30.84	0.07	0.79	20.54	0.00
DICQ – Social pressure to use	49.43	32.9	52.68	34.48	48.5	35.9	56.54	34.49	0.92	0.34	20.63	0.00
DICQ – Pleasant times with others score	59.14	27.5	63.49	28.64	67.5	27.02	68.68	29.12	0.00	0.99	13.93	0.00
DICQ – Overall score	56.08	26.8	61.76	26.14	61.06	25.14	65.73	28.2	0.22	0.64	21.68	0.00
Hepatitis-C Knowledge Questionnaire	16.22	2.29	16.2	1.79	17.44	1.89	17.61	1.66	0.08	0.78	16.46	0.00
Readiness to change stage	2.54	0.73	2.54	0.78	2.58	0.73	2.56	0.78	0.00	0.99	0.57	0.45

Scores on the HIV Risk-Taking Behaviour Scale similarly reduced for both groups, with the EPC group in particular exhibiting a large reduction in injecting behaviour which put them at a similar level to the SEC group, eradicating the difference that was present at baseline. Baseline scores on the HIV Risk-Taking Behaviour Scale did not account for differences on any of the outcome measures.

## Discussion

We were not able to demonstrate the efficacy of EPC compared to SEC in the prevention of hepatitis C amongst injecting drug users. The main reasons for this were the lower than expected levels of recruitment, coupled with the lower than expected compliance with the experimental intervention. The EPC and SEC groups were well matched in their demographic characteristics, drug use and psychological characteristics, including measures of risk behaviour. Levels of injecting equipment sharing behaviour were similar to that reported in other studies, with around 60% of all users who had injected in the past six months reporting sharing behaviour over the same period [5]. Of note is the difference in follow-up rates of SEC (56%) and EPC (77%) groups at 12 months. It is not certain whether this difference in follow up rate had any effect on the trial's internal validity and we have no explanation for this finding. Moreover these follow up rates do not correspond to retention rates in treatment as some of the participants had dropped out from treatment but agreed to attend for the follow-up research interview and HCV testing.

The difference in seroconversion was not significant between the two interventions at either six months or twelve months, but it was however in the anticipated direction, with fewer of those allocated to EPC seroconverting compared to those who received SEC. The difference was even more pronounced (but still not significant) when only those who received at least one session of the intervention were included as no patients who received at least one session of EPC seroconverted at either six months or twelve months. However, given the relatively low numbers of participants recruited and followed-up, and the differential rate of engagement in EPC and SEC and the even lower number of those who completed all 4 sessions of EPC therapy, no conclusions could be drawn and the efficacy of EPC in reducing new cases of HCV remains inconclusive.

Notably, there were many significant changes on some of the secondary outcome measures from baseline values, indicating positive change and improvement for both groups. These reductions in drug use and risk behaviour may reflect the impact of their treatment in general rather than any specific effects of the interventions. The finding that only half of the initial sample had injected in the last month at baseline, would suggest a broad treatment effect i.e. injecting behaviour had already stopped in half of the participants at intake. A recently reported RCT of a brief behavioural intervention in comparison with standardised educational intervention for reducing HCV risk practices among IDUs showed a reduction in these practices for both interventions at one month follow-up [27]. The study failed to demonstrate effectiveness of the brief intervention for a number of reasons: similarity between the interventions in duration and content, the short follow-up of one month and the inclusion of HCV positive IDUs.

It was also worthy of note that 60% of this high-risk group had never been tested for HCV prior to this research, despite the cohort being engaged at local community drug teams. In addition, 10% of those who had been tested in the past, and who believed themselves to be HCV sero-negative, were found to be HCV sero-positive, emphasising the need for regular testing of IDUs.

Possible reasons for the low overall incidence of 12.9 per 100 person years are the impact of treatment on risk behaviour [28], HCV screening and the provision of harm reduction approaches in the community. However there has been the notion that the impact of needle exchange programmes on the spread of HIV in IDUs has been limited in studies carried out in the US [29] and in Canada [30] with the conclusion that whilst needle exchange programmes are crucial for sterile syringe provision, they should be considered one component of a comprehensive programme including counselling, support, and education. Wright and Tompkins [31] in a systematic review of the evidence for the effectiveness of primary prevention interventions for Hepatitis C among injecting drug users reported that needle exchange programmes reduced the prevalence of Hepatitis C though prevalence remains high. However, methadone maintenance treatment was found to be only marginally effective at reducing HCV incidence and limited evidence evaluating the effectiveness of behavioural interventions in reducing its incidence. The review concluded that a robust response to the global health problem of HCV would require the provision of new behavioural interventions in addition to needle exchange and methadone maintenance programmes. Indeed one study [32] showed that in IDUs attending needle exchange schemes, brief intervention was effective in reducing alcohol use and probably attendant risk factors resulting in lower incidence of HCV in IDUs. Whilst the design of the present study has not enabled the examination of the contribution of treatment, needle exchange and HCV screening on the incidence of HCV, it is conceivable that both the SEC and the EPC interventions, in addition to the provision of treatment, together with the availability of needle exchange schemes for the present cohort of IDUs, has contributed to the low incidence rate of HCV in this population. Moreover, it is also conceivable that the testing regime instigated by the Research Team itself encouraged a change in risk behaviour: pre-test counselling in conjunction with the issues raised in the baseline interview, and a degree of self-selection. The impact of pre-test counselling alone is thus a potentially important mechanism of change in risk behaviour worthy of further investigation, and would indeed be a very encouraging development if it could be proven to be effective. In conjunction with the DBS, which has been shown to increase testing rates more than four-fold, the possibility of much greater testing taking place at primary care and other community settings, may help to monitor infection rates and help those who are not infected to remain so.

### Enhanced Prevention Counselling

The EPC intervention described here is one of the first such interventions developed specifically for the prevention of HCV with injection drug users. A process evaluation suggested that EPC facilitated a positive therapeutic alliance compared with the SEC control intervention and was perceived as beneficial by the IDUs in helping reduce HCV-risks [33]. The intervention was deliberately designed to be an enhanced counselling intervention as opposed to a Brief Intervention (BI) of one session only. A major difficulty, however, with the intervention was in attendance for treatment sessions; the majority of participants who engaged only attended for one EPC session. Thus in retrospect it appears that enhanced counselling is unlikely to be more effective than a single session BI as participants attend one session (at least in this study) regardless of what is on offer. Participants attended as normal for standard key working and therefore one implication may be that only one session is offered and any further work from this therapy programme might be better placed within standard key working.

A comparison with a group given no information or advice whatsoever is obviously not ethically possible and so the question as to whether any intervention, however brief, has any benefit (let alone knowing what the essential components of any intervention are) cannot be answered from the current study. From our clinical and field research experience, however, it seems likely that there are elements common to both interventions in this research that might be effective in helping prevent HCV infection. As with the Tucker [27] study, it is possible that the essential components of prevention in this clinical population is the time spent with the health professional and researcher, completion of the standardised questionnaires and particularly discussion of risk behaviours and the heightened awareness of risk this produced. The EPC may be made more fit for purpose by reducing the number of sessions from 4 to one or 2 sessions the first of which could be grafted on the post-HCV counselling sessions: this would ensure its higher uptake by IDUs and enable its evaluation. Moreover preventive behavioural interventions should be informed by reference to IDUs experience and views using qualitative methods such as focus groups.

### Methodological issues

The main problems encountered in the design, conduct and delivery of this research were the lower than expected levels of recruitment to the trial, and the low adherence to EPC but not the SEC. Retention of participants once recruited however was a lesser problem, as 65% of those recruited were followed-up, and adherence to the informational one brief session intervention was not a problem, as more than 90% of those randomised to the SEC informational intervention received it. The reasons for these two main problems can be grouped under issues relating to the participants' behaviour; issues relating to the service environment from which participants were recruited and issues relating to the trial design [6].

### Lessons learnt

One of the main lessons learned in this project is that conducting research in UK treatment settings presents challenges that are very different from those encountered in US studies upon which research in addiction is often modelled, as was the case in this project. RCTs in the US are usually conducted against a background of higher funding which facilitates pilot work, the formation of larger research teams, and therapists who are dedicated to the trial rather than relying on service staff trained in delivering the experimental and control interventions. Research in the US also benefits from a well-established clinical research infrastructure, which aids the introduction of new interventions, increasing compliance from staff and users. Indeed, the development and fostering of a culture of research within the services involved in the present trial was a task that had to be instigated. There is also reason to believe that the clinical populations in the US are different to those in the UK, with those engaged in treatment being older, and more socially stable; this is of consequence because it is important that service users are well-engaged in standard drug treatment regimes before introducing further demands such as structured counselling sessions. Another of the lessons learnt is the need for piloting of the new intervention in an area of research that involves the development of new interventions amongst a difficult clinical population, with only limited guidance available from other research.

One of the main policy implications for conducting trials of psychological interventions within addiction health care settings is for funding bodies to provide the necessary resources to improve the quality and comprehensiveness of treatment including the provision psychological interventions. This would provide the necessary infrastructure and capacity for the development of innovative interventions and thus offering opportunities for the evaluation of their effectiveness and cost-effectiveness in pragmatic clinical trials.

## Conclusion

We were not able to prove the efficacy of EPC in comparison with SEC in the prevention of hepatitis C in IDUs. Notwithstanding the negative findings of this research, we believe that the study has provided the benefits of developing and introducing specific behavioural primary preventive interventions (vide supra): the EPC and SEC interventions (manuals available from the authors) [33] and the DBS which hold promise and warrant further investigation. Moreover the main lessons learnt were that piloting of a new intervention is a crucial first step before conducting pragmatic RCTs of psychological interventions in the field of addiction; that an infrastructure and culture for psychosocial interventions is needed to enable applied research in the service environment, and research funding is needed for enabling the recruitment of dedicated trained therapists for the delivery of these interventions.

## Competing interests

The authors declare that they have no competing interests.

## Authors' contributions

MA was principal investigator, designed the study, had full responsibility for its overall management drafted and revised the article. PD designed, developed the manuals of the psychological interventions and trained and supervised the therapists. CD, NO, KC and HG contributed to the study design and methodology. PR, DM and CT were responsible for Hepatitis C testing and development of the Dried Blood Spot test. BC and CJ were the trial's co-ordinators and conducted statistical analyses under supervision of the biostatistician. CG was the health economist and designed the tools for measuring cost-effectiveness of the interventions. MD recruited participants from Surrey County. All authors reviewed drafts of the article and agreed the final manuscript and its revisions.
